# Ecology’s inattention to the city: Exploring a regime of scientific imperceptibility

**DOI:** 10.1177/26349825241241522

**Published:** 2024-04-22

**Authors:** Maud Chalmandrier, Valérie Boisvert, Joelle Salomon Cavin, Silvia Flaminio, Céline Granjou

**Affiliations:** University of Lausanne, Switzerland; University Grenoble Alpes, France

**Keywords:** Urban ecology, knowledge blind spots, imperceptibility, global, situated and everyday science, Switzerland

## Abstract

Promoters of urban ecology commonly point to the historical absence of the city in ecology. This assertion is obviously meant to highlight the novelty and timeliness of urban ecology and to plead for its development. Given the founding role of this ignorance narrative for urban ecology, we deemed it essential to explore whether and how it could be empirically substantiated. Drawing on ignorance studies, we propose to investigate knowledge blind spots and questions left uncharted by the dominant research agendas in ecology. Stepping aside from the shared assumptions within the urban ecology community, we set up to explore the main features of a regime of (im)perceptibility of the city in ecology. To this end, and using a mix of methods including bibliometric and textual data analyses, observations and interviews, we combined the exploration of global scientific publications, naturalist inventories in Swiss research institutions and cities and everyday ecological research practices in Switzerland. Our analysis leads to nuancing the binary representation of the city as either absent or present in ecological research. It highlights three dimensions (epistemic framings, field practices and institutional marginality) that may explain the imperceptibility of the city in ecological research. We demonstrate the existence of ecological research in the city before and alongside self-declared ‘urban ecology’. Ignorance studies generally aim to expose biased historiographies and address the politics of contentious knowledge. We hypothesize and show that this analytical framing can also shed light on the obfuscation of past and rival research in the formation and consolidation of epistemic communities.

## Introduction

In 1970, in his keynote address at the annual meeting of the American Association for the Advancement of Science in Chicago, the botanist Forest Stearns (1970) lamented that ‘biologists, with a few notable exceptions, have neglected the urban environment’ (p. 1006). This observation was echoed nearly three decades later by Herbert Sukopp (1998), a pioneer of the Berlin school of urban ecology, who wrote that: ‘For a long time, it was thought that urban areas were not worth studying with regard to ecology’ (p. 3). This statement has been reiterated by many other proponents of urban ecology (e.g. Gilbert, 1989; Grimm et al., 2008; McDonnell, 1997; Wu, 2014). It has generally served at once as a starting point and as a spirited plea for the recognition and development of the field, issued by authors proposing to contribute to it. However, while ecology’s earlier neglect of the city has been presented as a given, it has not in fact been thought through (Flaminio et al., 2023; Salomon Cavin and Kull, 2017). Although it has been pointed out by environmental historians and social scientists, including William Cronon, Mark Dowie and David Schlosberg (Light, 2001), the subject of ecology’s lack of interest in the city has featured mainly in the history of urban ecology as recounted by its main proponents and practitioners in support of their research agendas (Granjou et al., 2023). A core motivation of this historiographical endeavour within ecology itself is the aspiration to build and consolidate an epistemic community around urban ecology.

Conversely, some of the researchers who study urban environments – an undertaking whose history begins earlier than is suggested in this dominant historiographical treatment – do not identify with the label of ‘urban ecology’, reinforcing an invisibility already instituted by that historiography. At the start of the research project from which this article stems, we convened an international workshop on urban ecology in Lausanne.^1^ We had identified local biologists for a round table discussion, based on the alignment of their work with the topic of urban ecology. More specifically, we identified them based on our perception of their work in connection with urban ecology: that is, the perception of a group of social scientists, ‘outsiders’ with regard to the field of ecology. We were intrigued to discover that in presenting themselves they did not refer explicitly to urban ecology, but to their specific subdisciplines within ecology and objects of study, identified in terms that did not centre the urban dimension of their work (Chalmandrier and Granjou, 2021). Without actively denying their involvement in urban ecology, they simply did not use the term to represent their academic work, affiliation or stance.

The notion of the city’s absence from ecology is such a pivotal and recurrent motif in the foundational narrative of urban ecology (e.g. McPhearson and Maddox, 2018) that we felt that it was critical to delve into the ‘undone science’ in this field (Frickel et al., 2010; Hess, 2016).

In this article, our aim is to shed light on regimes of (im)perception or (im)perceptibility (Murphy, 2006) that may have, on one hand, (re)produced the lack of attention to the city in the ecological sciences, and, on the other hand and at another level, kept relatively long-standing practices of ecological research in cities invisible and the research results confined to the fringes of both mainstream scientific discourse and institutions. Our purpose is therefore neither to offer a synthesis of the ‘ecological constellations’ (Gandy, 2022) that have historically formed in relation to the plants and wildlife that thrive in cities (Alagona, 2022; Gandy and Jasper, 2020), nor to produce a genealogy of urban ecology as a field, nor to carry out a detailed study of some of its intellectual nodes, such as Berlin (Lachmund, 2013) or Baltimore (e.g. Grove et al., 2015; Kingsland, 2005). Our contribution to this special issue on ‘unknowing geographies’ aims to explore the widely noted historical inattention to the city in ecology and to shed light on research and practices that are imperceptible from the perspective of the dominant framing of urban ecology, having developed either *before* or *alongside* it. At a more general level, we intend to contribute to ignorance studies by offering new avenues of application. Drawing on our specific focus, we examine how historiographical narratives that render some topical issues and objects imperceptible, while exaggerating the originality or novelty of others, are integral to the disciplinary reconfigurations involved in the genesis of novel scientific fields.

We begin by presenting our sources of theoretical inspiration and explaining why we have chosen to approach the city’s place in ecology in terms of a regime of imperceptibility. We then survey the methodological challenges involved in capturing absence from a scientific field and introduce our approach. Our research is based on the analysis of several corpora and genres of publications, at different scales and from different standpoints, supplemented by observation of the everyday research practices of, and interviews with, ecologists in Switzerland. Since the country is not one of the recognized breeding grounds of urban ecology, we supposed that it would offer an alternative and decentred perspective. Through our analysis, we seek to provide a precise and nuanced understanding of the contours of inattention to the city in ecology. To do this, we identify a regime of imperceptibility based on three dimensions: epistemic framings, field practices and institutional marginality.

## From knowledge blind spots to regimes of imperceptibility

Scholarship on ignorance has expanded considerably over the past few decades. A new research field dedicated to it, agnotology, has emerged (Kourany and Carrier, 2020; Proctor, 2008; Proctor and Schiebinger, 2008). Agnotology is concerned with the diversity of forms and states of ignorance – whether unconscious or intentional, passive or active – produced and sustained by an interweaving of epistemic framings, practical research contingencies, histories, spatialities and political and cultural contexts. Agnotology illustrates the cumulative effects of the multiple selectivities of research, revealing untrodden paths in various scientific fields (Hess, 2007).

In geography in particular, the significance of ignorance is increasingly being pointed out (Birkenholtz and Simon, 2022; Gross and McGoey, 2015). Most geographical studies in this vein fall within the realm of political ecology and accordingly emphasize the active and deliberate production of ignorance. With a view to challenging established knowledge, ignorance research focuses on the political uses of ignorance and on how such uses reinforce the status quo, naturalize power relations and tend to present dominant positions as neutral and scientific. For example, it has highlighted racialized ignorance (Mills, 2007), how colonial science has obscured indigenous knowledge (Vitebsky, 1993), how gender biases have blocked or limited certain lines of inquiry (Schiebinger, 2004; Tuana, 2004) and how knowledge was manipulated or censored for political purposes during the Cold War (Oreskes and Conway, 2008) and, more recently, to support post-truth politics (Simon, 2022). The collusion between science and industry in producing ignorance about matters that touch on the interests of powerful economic sectors has often been highlighted from a critical perspective (McGoey, 2012, 2019; Oreskes and Conway, 2010). Among the frequently cited examples are research on pesticides (Shattuck, 2021a, 2021b) and dying bees (Kleinman and Suryanarayanan, 2013).

Work undertaken from this type of perspective primarily strives to highlight the production of ignorance or the maintenance of knowledge deficiencies as a deliberate strategy (Proctor and Schiebinger, 2008) or as a ‘resource’ to ‘increase ambiguity, cause controversy and/or delay action’ (Birkenholtz and Simon, 2022: 157). It aims to expose the entanglement of scientific and political claims, the internalization of dominant ideologies or the colonization of research imaginations by culturally embedded beliefs. It reassesses science and historiography, seeking to reveal standpoints and relativize modern science’s claims to universality.

Another strand of scholarship considers what Birkenholtz and Simon (2022: 156) call ‘ignorance as outcome’, looking to capture the emergence of ignorance as an unintended side effect of research agendas that are themselves intentional. Hess’s (2016, 2020) notion of ‘undone science’ is relevant here. Initially, he identifies it as research that has not been carried out or that remains marginal or limited in scope in comparison with priority questions that crystallize research resources and efforts, and in relation to which methods and evaluation criteria are calibrated. In this case, certain questions are not asked and certain objects are not addressed because they fall outside the dominant frame and are invisible from the point of view of the prevailing epistemic norms and scientific orthodoxies (Hess, 2016). However, Hess (2020) also points out that undone science can occur when ‘research is designed in a way that precludes some categories of data collection and research questions because of methodological preferences’ (p. 243). In particular, this can result from analytical knock-on effects of regulatory science and its ‘epistemic form’ that lead to compounding and accumulating ignorance. Proctor (2008) differentiates ‘ignorance as native state’, which ‘implies a kind of deficit, caused by the naiveté of youth or the faults of improper education – or the simple fact that here is a place where *knowledge has not yet penetrated*’ (p. 4) from ‘ignorance as lost realm’ (or ‘selective choice’, or passive construct), a formulation he uses to underline that inquiry is always selective (pp. 6–7). As he notes, ‘ignorance is a product of inattention, and since we cannot study all things, some by necessity – almost all, in fact – must be left out’. He also notes that while ‘selectivity is often conceived as transient, evanescent, a kind of “noise” in the system or scatter about the line, with bias slowly being rectified’, in fact, ‘knowledge switched onto one track cannot always return to areas passed over. . . . Research lost is not just research delayed; it can also be forever marked or never recovered’ (Proctor, 2008: 7).

The urban ecology scholars who point out or decry the scant attention paid to the city in ecology do not produce a grand teleological narrative where the ecological sciences consign the city to a strategic oblivion on economic or political grounds. When they expand on their initial assertion of ecology’s inattention to the city, they argue that it stems from a blind spot in ecological science, which was built around the study of relatively untouched ‘natural habitats’ (e.g. Botkin and Beveridge, 1997; Trepl, 1996).

Some scholars, such as the Berlin ecologist Ludwig Trepl (1996), suggest that ecology has long been an ‘anti-urban affair’ (p. 89) that embraces a romantic vision of nature associated with a condemnation of the modern city. Stearns (1970: 1006) points out that the complexity of the city and its specificity as an ecosystem may have put ecologists off. For Sukopp (1998: 3), this disdain stems from the view that cities are ‘anti-life’ from a biological viewpoint. Such representations of cities and nature as counterpoints to each other (Castree, 2003; Gandy, 2006) reflect modernity’s characteristic conceptual dualism between nature and culture (Latour, 2005). However, the explanatory scheme offered by the grand narrative of modernity is so general that it scarcely seems suitable as a guide for empirical investigations aimed at shedding light on science in the making.

In his ‘ecologist’s perspective’ on the history of urban ecology, McDonnell (2011) associates the urban blind spot in ecology with the equilibrium paradigm, which he argues ‘implies that to effectively study “nature” . . . ecologists had to locate study sites far from human actions’ with the direct consequence that ‘for much of the twentieth century the discipline of ecology contributed relatively little information to our understanding of the ecology of human settlements’ (p. 7). Human settlements, including cities, are obviously not among the environments that have served as archetypes in the historical development of the discipline, such as forests, lakes or islands (Drouin, 1993). The habit of not taking cities into consideration presumably led to current practices and methods in ecology that reinforce this exclusion and produce invisibility. Until recently, while some ecological research may have taken place in cities, ecologists did not routinely consider the city itself as an object of knowledge or theorization (Flaminio et al., 2023). They were committed to agendas and definitions of the ‘knowledge that matters’ (Granjou and Arpin, 2015) that did not include urban space and objects as such. Thus, even the proponents of urban ecology do not present the historical neglect of the city in ecology as a deliberate resource or strategy, but as an effect of research practices and agendas, dominant epistemologies and associated imaginaries and local contingencies.

This representation resonates with the notion of ‘inscrutable spaces’, defined by Kroepsch and Clifford (2022) as ‘spaces that are made difficult to know by an interplay of biophysical, epistemic, and political economic factors, and whose unintelligibility poses serious consequences for environmental politics and everyday life’ (p. 17).

It also echoes the concept of the ‘regime of (im)perceptibility’, defined by Murphy (2006) in her exploration of sick building syndrome as ‘the way a discipline or epistemological tradition perceives and does not perceive the world’ (p. 10). Murphy (2006) points out that from this perspective, ‘what counts as truth is the result of historically specific practices of truth-telling – laboratory techniques, instruments, methods of observing, modes of calculating, regimes of classification, and so on’ (pp. 7–8), emphasizing the implication that ‘other, yet undeveloped, ways of registering, slicing up, and bringing into being the complexity of the world are, were, and will be made possible by new instruments, techniques, social movements, and so on’ (p. 8). She thus sets out to uncover the singular historical assemblage that has enabled issues or objects to assume materiality, to become detectable and actionable, with a view to capturing what previously made them imperceptible.

We feel that this approach, and the core concept on which it is based, are highly relevant to our subject matter. We therefore propose here to explore and characterize in this light the regime that produced ‘historically specific terrains of invisibility’ (Murphy, 2006: 111) and made the city such an inscrutable space for ecology.

## Methodology and material

The empirical study of ignorance raises logical and practical obstacles, ‘since, by definition, it exists [only] in a negative sense as an absence or near-absence’, as the reverse side of presence, as pointed out by Frickel and Kinchy (2015: 180). While it is comparatively easy to identify and then study a topic that is present in a scientific corpus, to map the associated networks or to report on controversies, dealing with absence is more uncertain, uncomfortable and conditional. This holds especially when knowledge blind spots are not deliberately produced and maintained. Strategic ignorance can be approached by exposing the power struggles and interests it serves and the strategies used to prevent certain matters from arising. Inadvertent ignorance that emerges incidentally as a side effect of deliberate choices of research topics or questions is more elusive.

### Tracking ignorance in practice

Ignorance is a fundamentally relational and subjective notion. The absence of a subject can only ever be asserted in reference to a given corpus, space or epistemic community. It is transient in nature and can only be argued until the contrary is demonstrated – until a field, category of writings or experiences that counter this argument is identified (Murphy, 2006).

As mentioned above, ignorance studies have largely developed with a view to uncovering the biases that have marked the constitution of scientific fields and the pressures that prevent certain questions from being addressed. It highlights the links between dominant scientific agendas and the preferences of economic and political elites, which can lead certain issues to remain unexplored (Frickel et al., 2010). Conversely, it often looks at social activism as a powerful revealer of scientific blind spots. According to Hess (2007), social movements, industry reformers and civil society organizations, such as patient groups and other mobilized publics, are instrumental in exposing ignorance in areas that should be investigated in the name of the general interest, the environment or public health. This observation cannot readily be transposed to urban ecology. Urban ecologists frequently raise the issue of the unsuitability of certain ecological approaches to human-made ecosystems, and the consequent inadequacy of the resulting knowledge to guide urban planning. Urban policies are of course sites of contestation, for example of ‘New Urban Science’, which relies on ‘the application of big data, ubiquitous sensing, geospatial and social network analyses, algorithms, machine learning and artificial intelligence’ (Karvonen, 2020: 418). Some call for bottom-up modes of urban science involving ‘citizen sensing initiatives, living urban laboratories, and other democratic modes of situated knowledge production that are designed, developed, and executed by civil society organizations and neighborhood groups’ (Karvonen, 2020: 418–419). However, identifying a large-scale social movement or broad coalition of urban ecology activists whose knowledge and demands have the potential to shed light on key issues requiring scientific attention poses a daunting challenge. The pivotal factors at play revolve around the politics of science and knowledge. They are more difficult to pin down in empirical terms.

In order to define the incompleteness of an approach, a vision of a fuller and more appropriate way of dealing with the subject is needed. Among the many issues left aside by mainstream science, those that come to be labelled as ignored are those that eventually arouse the interest of scholars who judge in retrospect that they should have been addressed earlier or those that emanate from peripheral, marginal or subaltern authors or currents.

Past ignorance raises specific additional challenges. It can only be characterized by looking for the present in the past – and not finding it there. This amounts to producing a genealogy of the present that sets aside the singularities of time and place, seeking ‘precursors’ and ‘premonitory intuitions’ in the past and framing them as milestones of an evolution that, in the light of historiographical assessments strongly oriented by this ‘view from the present’, is seen as culminating in contemporary ecology. As Walter (2014) points out, ‘most innovative writings leave no descendants, until they are instrumentalized’ (p. 28) as part of such a misleading genealogy. The same critical point applies to ignorance, which can only be characterized as such in the light of current research interests and agendas. And yet, as Shapin and Schaffer (1991) argue, ‘description of past science could be distinct from celebration of present-day science; its interpretation could be distinct from identifying its role as “foreshadowing” modernity’ (p. xxi).

Although we share an interest in nature in the city, we are all social scientists and hence cannot report from the inside on developments in ecology and conservation science as disciplines. Our ambition is neither to endorse nor to discredit the narrative of the absence of the city in ecology, but simply to give a different account of it. We are faced with the practical challenge of developing what Shapin and Schaffer (1991: 4) call a ‘stranger’s account’ (as opposed to a ‘member’s account’), which involves stepping aside from the shared assumptions, framings and scripts within the urban ecology community. This leads us to approach the absence of the city in ecology on the basis of a range of data and observations, without unreservedly adhering to the dominant narrative of ecology’s recent encounter with the city, by collecting situated testimonies and bringing to light the contexts and attachments they reflect.

### Exploring ignorance across scales: Ecology as science, context and practices

Recognizing the importance of the epistemic specificities of ecology and of the subjectivity, spatiality and diversity of researchers’ lived experiences, we adopt a multi-scale approach, based on the exploration and articulation of different sets of data from different periods.

Guided by approaches to ignorance in other subject areas, in order to capture and characterize the place given to the city in ecology, we seek to view ecology in a broad sense and to make use of a multiplicity of scales and angles of observation and analysis (see Table 1). We therefore understand and approach ecology at once as a scientific discourse codified within global epistemic communities, as a discipline instituted within national academic worlds and their institutions, and as a set of lived experiences and everyday practices. One of the best ways to avoid producing a totalizing and universalizing discourse on the city in ecology is to avoid essentializing the discipline and its objects of study, instead examining it in context and in practice.

**Table 1.** Three scales and angles of analysis.

	Type of sources	What the data can reveal	What the data cannot reflect
Global science	Articles from leading journals in ecology and conservation science Member directories or abstracts and proceedings from international ecology conferences (e.g. ESA conference, GfÖ) Funding agency databases of international research projects	Disciplinary canons and norms defining the ‘science that matters’ Dominant research actors and institutions Places, thematic trends, epistemic communities, research networks	The practical, material and contextualized conditions of knowledge generation The political, social and economic dimensions of research activities Everything that remains implicit in the publications (e.g. tacit norms shared and incorporated by researchers; aspects that escape the standard formatting of scientific publications)
Situated knowledge	Journals, bulletins and various publications of local natural science societies Reports produced by ecological consultancies Publications of community groups, local NGOs Regulatory science University curricula in ecology . . .	Regional/local ecological research production sites, actors and institutions Scholarly works in languages other than English and/or in formats other than international scientific publications (e.g. reports, handbooks, guidelines), that do not always circulate internationally. Alternative, anonymous, marginalized forms of knowledge The context of research production (e.g. links with policy making)	Major international trends in the discipline Forms of ecology in other national/local contexts
Everyday research practices	Interviews with researchers Case studies Ethnographic observations, including participant observation (e.g. laboratory life, field practices) Unpublished papers, personal archives (e.g. activity reports, minutes of meetings, laboratory notebooks, field notebooks)	Science in practice and context Biographical trajectories, job switches (e.g. from public research institutions to private consultancies), relationships between researchers Field and laboratory life Micro-events, changes that affect the life of the researcher (e.g. conflicts or affinities between people, material contingencies)	Major international trends within the discipline Risk of a magnifying-glass effect and a reality distorted by a situated experience

We thus look at (i) the established and dominant global scholarship as reflected in English-language publications in international peer-reviewed scholarly journals, (ii) the activities of local naturalist societies in Switzerland, reported in German or French in their publications as expressions of situated research traditions in ecology and, finally, (iii) the trajectories of ecologists in Switzerland within and outside the academic world in the 1990s.

No single angle or level of analysis is sufficient on its own. It is the combination of internalized disciplinary norms, research policies and cultures materialized in academic institutions and field practices that ultimately determine research agendas and questions and that may lead some topics to be privileged and others avoided.

Scientific publications only capture certain types of research and knowledge production. Not all accumulated practical knowledge and material research practices are recorded in this way. For instance, ecological surveys conducted for regulatory purposes and activities such as teaching, training and public awareness initiatives contribute to local knowledge generation and sharing.

We draw on the example of Switzerland to illustrate the possibility of place-specific research configurations. These can be addressed by studying the local arenas in which ecological research is produced and used in policy making, and the culturally specific routines governing them. We chose Switzerland for several reasons. It is a practical choice since most of the authors are based there, but it also occupies a median position with respect to our theme, somewhere between the acknowledged pioneering places of urban ecology and regions where the field remains either marginal or less widely publicized. There are no self-proclaimed or recognized centres of urban ecology in Switzerland comparable with Berlin (Lachmund, 2013) or Baltimore (Grove et al., 2015; Kingsland, 2005).

Building on these methodological premises, we performed a two-stage analysis, consisting, first, of identifying references to the city over time in two corpora (articles from international scientific journals and publications from local societies), and, second, of characterizing the forms of (in)attention to the city that we identify in the process.

### Situating the presence/absence of the city in ecological literature

Many of the bibliometric literature reviews used to demonstrate the development of urban ecological research beginning in the 1970s are the work of practicing ecologists (e.g Barot et al., 2019; Collins et al., 2021; Magle et al., 2012; Rega-Brodsky et al., 2022; Young and Wolf, 2006). These reviews identify the main themes and approaches in the field, its geographical focus and research gaps. Whether they highlight how under-investigated urban ecological research is (Collins et al., 2000) or its current dynamism (Muderere et al., 2018), these reviews seek to legitimate urban ecology, to bring together dispersed research work and to propose a joint research agenda. Our analysis differs from them in that it is based on a larger – and, to some extent, qualitatively different – corpus, covers a longer period. It is intended neither as an apologetic for, nor a critique of, urban ecology.

The first stage of our analysis aimed to highlight the presence of the city^2^ – and correlatively its absence – in the ecological literature, and was conducted on two textual corpora.

The first corpus was compiled from 10 international journals in ecology (*Ecology*, *Journal of Ecology*, *Journal of Animal Ecology*, *Oikos*, *Ecological Monographs*, *Journal of Applied Ecology*, *Oecologia* and *Trends in Ecology and Evolution*) and conservation sciences (*Biological Conservation* and *Conservation Biology*). They were chosen for their importance in ecology – they are among the most cited journals – and for their age – the oldest, the *Journal of Ecology*, was created in 1913. This choice is in line with our intention to look for references to the city in ecology before the advent of urban ecology and outside the journals explicitly dedicated to it. This corpus, which runs from 1922 to 2018, allows us to record the presence/absence of the city over a relatively long period. A total of 960 articles dealing with cities were identified. This was done by systematically searching for words such as ‘urban’, ‘city’ or ‘suburb’ in the abstracts, keywords and titles of the articles using a cross-search of JSTOR, Web of Science and Science Direct. Irrelevant articles were extracted manually (e.g. when the only city name cited was that of the author’s home university).

The second corpus was made up of the publications of nine French- and German-language Swiss cantonal^3^ natural science societies (Basel-Stadt, Basel-Landschaft, Bern, Fribourg, Geneva, Luzern, Neuchâtel, Solothurn and Vaud) and the national journal of the Swiss Botanical Society, between 1839 and 2018. This choice was guided by criteria of age, online accessibility in the E-Periodica database and the representation of different spatial contexts (predominantly rural or urban) and language regions.^4^ Natural science societies have long contributed to inventories of flora and fauna and have accumulated archives that were easily accessible to us in German and French, thereby sidestepping the hegemony of English as a scientific language. Comprising both professional and amateur naturalists, they are historically key sites for the organization of naturalist scholarly activity in Switzerland (Scheidegger, 2017). In Switzerland and more generally, their members contributed to the institutionalization of disciplines like botany and zoology and to the development of ecology from the end of the 19th century (Alberti, 2001; Benson, 2000; Lowe, 1976). With the professionalization of science during the 20th century, amateur naturalists were gradually excluded from academic knowledge production, and academics increasingly turned away from regional natural science societies’ journals for publication of their results. We applied a trilingual German-French-English search in all article texts for the words stadt*, städt*, ville*, agglo*, urbain*, urban*, *city* and *cities*. A total of 444 articles containing occurrences of these terms were manually selected and coded (see methodological Supplemental Appendix).

### Characterizing imperceptibility

The second stage of analysis aimed to characterize in greater detail the context and modalities of reference to the city in the corpora. This involved answering the following questions for the selected texts: What is studied in the city (themes, species, sites, etc.)? How is the city qualified, defined and represented? What ideas is the city associated with? Does the city constitute a research object in its own right, or is it simply the location of research whose central object is elsewhere? Does the article have an empirical focus or does it have more theoretical ambitions? To answer these questions, we performed textual data analyses on the corpora mainly using the open-source programme TXM (Heiden et al., 2010) complemented by a thorough reading of a large number of texts. For both corpora, these analyses required a significant amount of coding (see methodological Supplemental Appendix). In the Swiss corpus, the practices of naturalists were analysed in depth and in context, in relation to the social and material dimensions of the city and their entanglements in locally embedded scientific communities.

We also carried out a survey on the conditions of production of ecological research on the city in Switzerland. This survey was primarily based on semi-structured interviews with actors conducting ecological research on the city (mainly academics, but also consultants and city government employees). The interviews focused on their life stories – their beginnings in urban ecological research and the role it played in their career; their research experiences in the city – the type of projects they have been involved in and their approaches, methods and fieldwork practices; their networks and their status, as reflected by institutional and financial support. This allowed us to situate the experiences, activities and engagements of ecologists in the hierarchies of recognition and status within Swiss academia, as indicated by the resources allocated to urban ecological research. To do so, we combined a set of heterogeneous institutional sources on Swiss ecology, such as nationally funded research projects, descriptions of professorships in universities and research institutes’ activity reports.

The combination of a systematic diachronic exploration of textual corpora and more qualitative research on key sites of the emergence of urban ecology in Switzerland allowed us not only to characterize relations to the city as an object in the dynamics of knowledge production (by looking at framings, practices, methods and discourses) but also to assess the field’s scientific importance by characterizing the scientific legitimacy and recognition of the researchers who work in it.

## Results

Our analysis highlights different types of ‘knowledge blind spots’ in relation to the city in the ecological sciences. We first identify references to the city in the corpora and trace their evolution, which allows us to quantify the absence of the city, albeit in a somewhat crude fashion. We then seek to highlight and characterize the processes that explain and perpetuate the imperceptibility of the city in ecology.

### The city: A significant absence in ecology?

**Figure 1.**
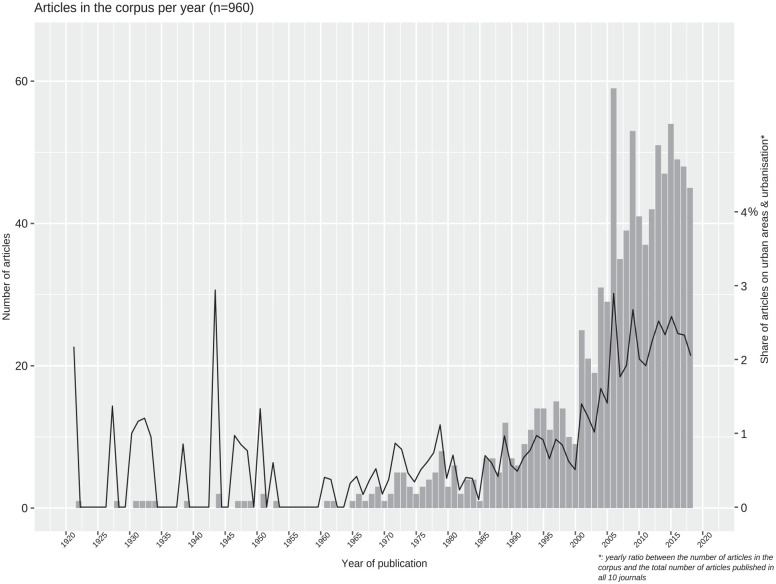
Articles on cities in the top 10 ecological journals, in absolute numbers and as a proportion of the total number of articles, 1922–2018. *Source*: Modified from Flaminio et al., 2023.

In the international corpus, the city is largely invisible before the 1960s. The number of scholarly publications mentioning urban areas or urbanization never exceeds three per year, with years without any publication (Figure 1). The number then increases, but the percentage of publications in the ecology and conservation journals remains very low. Publications on the city in our international corpus account for only 0.6% of the total number of papers published in the 10 journals between 1970 and 2000; in the 21st century, the annual percentage ranges between 0.5% in 2000 and 2.6% in 2007. These results fully confirm those of comparable studies carried out on slightly different corpora and periods (Collins et al., 2000; Collins et al., 2021).

The near-absence of mention of the city in scholarly publications in ecology is even more pronounced if we exclude the two conservation science journals, *Biological Conservation* and *Conservation Biology*, founded in 1968 and 1987, respectively. These two journals together account for more than half (57%) of the publications mentioning the city in the corpus. This observation highlights how the development of a problem-oriented ecology, dedicated to the management and conservation of natural resources and habitats, has fostered ecology’s interest in cities. The management of human-modified ecosystems, especially urban-industrial systems, also became a key aspect of the international Man and Biosphere Programme (MAB) established in 1971 by UNESCO (Batisse, 1971). It was further strengthened by the advent of conservation biology, which contributed to putting the biodiversity crisis on the international agenda and reinforcing the influence of scientific and expert-led approaches to conservation politics (Takacs, 1996).

Our analysis therefore puts the discourse of ‘exponential growth’ in publications on cities in ecology in recent years into perspective (e.g. Barot et al., 2019; Muderere et al., 2018). While this growth has indeed been impressive in absolute numbers, it can only be interpreted with reference to the most salient development of recent decades, which is an explosion in the total number of scientific articles and the amount of science-related digital data (Fortunato et al., 2018).

**Figure 2.**
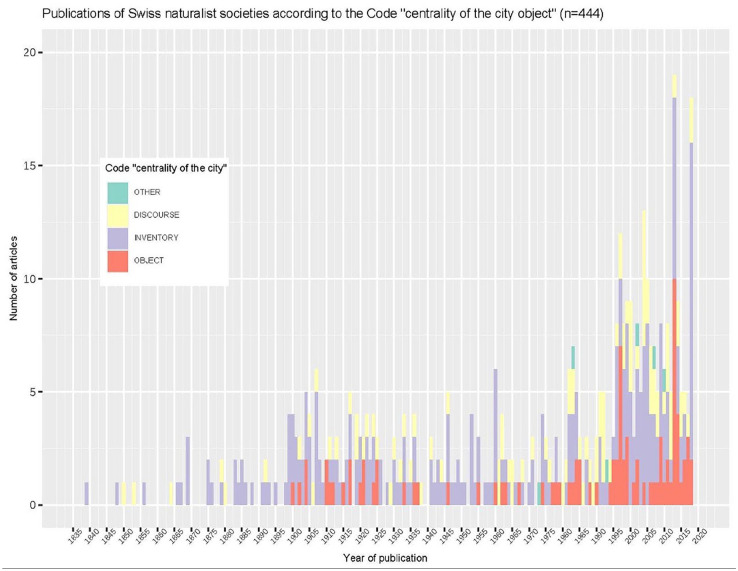
Publications mentioning the city in Swiss naturalist society journals, in absolute numbers. As the corpus is a broad selection of publications mentioning the city, a code was applied to classify them according to the centrality of the city as a research object. ‘Discourse’ refers to publications which contain general discourse on the city or urbanization without reporting field research in cities. ‘Inventory’ publications mention species collected at least partly in the city or its immediate surroundings, without containing any discourse on the city and its non-human inhabitants. ‘Object’ publications present fieldwork carried out at least partly in cities or their immediate surroundings and discourse on non-human beings in cities. The graph shows only absolute numbers of publications, not percentages of the total number of articles, as the journals of the natural science societies in the corpus also cover other disciplines such as physics, chemistry and geology (see methodological Supplemental Appendix).

Our analysis of the corpus of Swiss naturalist society publications reveals a similar trend, albeit with a time lag. Insofar as the period covered by the analysis is longer, it also allows a slightly more nuanced picture to emerge. During the 19th century, naturalists almost exclusively botanized in the outskirts of cities rather than in their central districts (see also Mathis and Pépy, 2017: 220). The first decades of the 20th century saw an increase in the number of floristic and faunistic inventories partly carried out in cities. This interest then declined until the 1980s, when the total number of publications including the number of ‘object’ publications (see Figure 2) increased, as local inventories began to focus on the city itself, and urban areas were more systematically explored as part of regional inventories.

The slight differences between the findings from the two corpora can be explained by the different emphasis placed on naturalist practices within them. While the research of naturalists is often confined to the ‘protohistory’ of urban ecology and is absent from the history of its recent developments as reported in leading ecological journals, the regional corpus highlights the ongoing presence of naturalist activity in Switzerland and raises the question of its contemporary role in urban ecology.

The near absence of references to cities in academic publications is an unambiguous sign that the city is not a research focus for their authors. Further investigation is needed to move beyond this simple observation and attempt to account for a possible regime of imperceptibility of the city in ecology.

### Beyond absence: Forms of imperceptibility of the city

We highlight three dimensions that contribute to the prolonged imperceptibility of the city in ecology, even when the city (or some of its portions) is a research location. The first is the enduring prevalence of framings and theories that have not formalized the city as a significant spatial and epistemic category. The second, exemplified by the research of naturalists, is the predominance of field practices implemented at scales other than that of the city as a territorial unit – whether larger or smaller. The third and last is the marginal status of urban ecological research and researchers, who are confined to the fringes of the academic system.

#### Cities as unseen backdrops

Historically, in many studies, cities incidentally happened to be the physical sites of research focused on something else, most often species (e.g. a census of house martins and swallows near Manchester: Cramp and Ward, 1934). Cities were thus presumably not caught up in what De Bont and Lachmund (2017) term the ‘spatio-epistemic processes’ (p. 7) of ecology: Their social and material characteristics did not influence the field’s scientific productions, nor were they in turn shaped or redefined by the framings of its researchers.

From the 1970s onwards, a greater number of articles in ecology publications were devoted to characterizing the city in relation to its periphery. The emergence of new ecological approaches may have been instrumental in this respect, by providing new conceptual resources, techniques and spatial models. This was the case with landscape ecology, which deals with the spatial and temporal dynamics of the biological, physical and social components of anthropized and natural landscapes. Within this analytical framework, the city was conceived as a stage in an urbanization gradient (McDonnell and Hahs, 2008; Ter Braak and Prentice, 1988), and urbanization was described as contributing to the heterogeneity and fragmentation of ecosystems, with effects on species distribution, diversity, richness and abundance (Flaminio et al., 2023).

**Figure 3.**
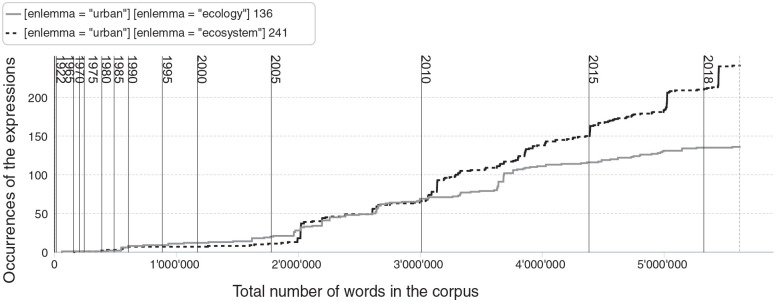
Cumulative frequency of the expression ‘urban ecosystem’ and ‘urban ecology’ in the corpus. The figure illustrates how the two expressions first appeared in the early 1980s and how their usage mostly increased after the mid-2000s.

In line with these developments, recent articles have more frequently included discussions of the conceptualization of the city as an ecosystem (Figure 3) or socio-ecological hybrid, as well as definitions based on land use (Gaston et al., 2005), minimum coverage of hard surface (Bonnington et al., 2013: 16), building density (Møller, 2009: 851) or human population density (Shochat et al., 2006: 186). The expression ‘urban ecology’ has also become increasingly common, highlighting how researchers recognize their work as falling under this label even in generalist ecology journals (Figure 3).

#### The influence of local naturalist practices

In the corpus of publications of Swiss local natural science societies, the absence of the city at the turn of the 20th century is an effect of the practices of naturalists (Figure 2). Swiss naturalists focused on characterizing local natural diversity, aiming at listing species on a scale that can be loosely defined as that of the cantonal region. In this context, occasional references to cities as collection sites reflected the growing popularity of local outdoor inventory practices (Kohler, 2006; Scheidegger, 2017), rather than the change in the status of urban observations in the geography of naturalist activity.

However, in the first decades of the 20th century, a particular type of urban naturalist practices experienced a temporary craze, before declining from the 1930s onwards. They were linked to the study of adventive plants and ruderal flora, which arrived in cities with the construction of railroads. A network of amateur and professional botanists embarked on the careful exploration of railway ballasts, municipal landfills and industrial areas (e.g. Cruchet, 1933; Nägeli and Thellung, 1905; Probst, 1914). Fascinated by exotic spontaneous flora, they produced rich descriptions of the physical characteristics of these places and associated human activities, highlighting their relationship with industrialization, urban development and international trade. However, the geographical imagination of naturalists did not extend to the whole urban space, which they did not conceptualize as such.

Interest in urban naturalist practices was revived in the 1980s with the first urban flora projects in several Swiss cities (e.g. Brodtbeck et al., 1997; Brun-Hool, 1994; Landolt, 2001). They marked a significant shift in scale compared with older botanical inventories: On one hand, they focused on the city per se; on the other hand, the spatial resolution of field practices was much more precise. They aimed to explore every corner of the city and therefore spanned several years. As inventory sites diversified and the composition and distribution of plant species were mapped, ecological representations of urban space were reordered (see also Lachmund, 2004).

Based on field practices that resonate with the naturalist culture of the Berlin school of urban ecology, the city was redefined as a biotope-mosaic and claimed as an ecosystem in its own right. The ‘discovery of the city’ became a recurring motif in botanists’ pleas for greater recognition of the city’s role in conserving biological diversity, as a valuable habitat for wildlife (Chalmandrier et al., 2023).

#### Urban ecology: A fringe science?

Our study shows that in Switzerland, urban ecological research, which emerged in the 1980s–1990s, remained peripheral and imperceptible at the end of the 20th century in two senses: vis-à-vis national academic institutions dealing with ecology, and within European urban ecology.

The first urban ecology studies were mainly carried out by groups of young academically trained biologists who were actively involved in environmental movements. Their collective aspiration was to put their scientific activity at the service of an expert-led nature protection. As biology curricula and academic research in Swiss universities made little room for applied and field ecology, these young biologists produced ecological knowledge on cities without funding or in their spare time (Brodtbeck et al., 1997; Ineichen, 1997). Most opted for academic activism and a career on the fringes of academia as consultants, either without a doctorate or during or after completing a doctoral thesis (e.g. Gloor et al., 2001; Wilhelm, 1997). Most eventually managed to develop applied ecological research in collaboration with city and cantonal administrations with an interest in urban nature conservation.

Ecological research on cities within universities was rare and never played a pivotal role in academic careers, as illustrated by the case of Elias Landolt (1926–2013). Professor of geobotany and director of the Geobotanical Institute of the Swiss Federal Institute of Technology (ETH) in Zurich from 1967 to 1993, he developed an interest in the city as a late venture in his career. In 1984, he started a major long-term project, *Flora der Stadt Zürich* [flora of the city of Zurich] – botanizing mainly in his spare time – which he completed after his retirement (Landolt, 2001). To carry out his personal urban flora project, Landolt created a group dedicated to urban ecology (‘*Stadtbioökologie*’) within the institute and supervised a dozen diploma students and two doctoral students.^5^ They surveyed and mapped plant communities across diverse urban land-use types, analysed their ecological requirements and the influence of human activities and proposed management practices likely to enhance the biological diversity of green spaces. During its short existence, from 1988 to 1995, the group was very active, but precarious, as Landolt, close to retirement, was unable to support it financially. Many former students set up their own ecological consultancies, while urban ecology, as an isolated and ephemeral interest, disappeared from the research orientations of the Institute with Landolt’s retirement.

The trajectories of Swiss ecological scholars, and their lack of institutional resources, are intertwined with the dynamics of ecology. Ecology has long been a marginal discipline in Switzerland, caught between the domination of functional and molecular biology and the decline of forms of ‘organismal biology’, such as systematics (Benz, 2019; Stettler, 2002). However, by the end of the 1980s, several scandals linked with environmental disasters and the emergence of the biodiversity crisis as a public issue prompted universities to develop training and research that would address environmental issues (Gisler, 2020). Ecological biology was institutionalized, with a surge in the creation of new professorships and research groups in ecology and an expanded place for ecology in environmental sciences curricula. However, the academic development of ecological sciences in Switzerland did not initially favour urban ecology. On one hand, it led to a shift towards novel approaches to biodiversity at different levels (genes, species, communities) and its functions in ecosystems. In parallel, more conventional approaches to biological diversity, such as systematics and phytosociology, came to be regarded as obsolete. As these approaches formed the historical basis of field research at Swiss universities, this accentuated the separation and hierarchical division between basic and applied knowledge within the emerging field of conservation biology. The rising generation of university ecologists was not interested in the urban environment, while urban ecologists who were not affiliated with academia devoted themselves to the production of knowledge whose scholarly recognition was limited.

This configuration seems to be specific to Switzerland. In Germany, Belgium and the United Kingdom, far from being an obstacle, the involvement of urban ecologists as experts in urban planning has, on the contrary, been a powerful lever for the development of the academic field of urban ecology. Paul Duvigneaud in Brussels (Danneels, 2023) and Herbert Sukopp in Berlin (Kowarik, 2020; Lachmund, 2013) are illustrious examples. Similarly, in Great Britain, urban wildlife ecology has developed in close collaboration with both governmental and non-governmental wildlife protection organizations (Adams, 2005).

The nature of urban ecology’s research agenda is probably not the reason why it was marginalized in German-speaking Switzerland in the 1980s and 1990s. This research agenda drew very directly on acclaimed conceptual and applied research conducted at German universities, notably Kassel, Dresden, Saarbrücken and above all Berlin. Swiss researchers also had occasional exchanges with Germany: literature sharing, short research visits, invited lectures and conferences. However, since urban ecology emerged in German-speaking Switzerland a decade later than in Germany, Swiss researchers did not attend the founding events of urban ecology in Europe, such as the 1980 European Ecological Symposium dedicated to the field (Bornkamm et al., 1982). In Brussels and Berlin, urban ecology was supported and developed by prominent academics in this period, enabling their respective projects to benefit from early support and institutionalization. The Centre for the Study of the Urban Environment at the Université libre de Bruxelles, and the Institute of Ecology at the Technical University of Berlin, whose fieldwork focused on the urban environment of West Berlin, were both established in 1973 (Danneels, 2023; Lachmund, 2013). Different local configurations in Switzerland have thus precluded the organization of a distinct scientific community around the production of knowledge in urban ecology.

## Conclusion

Our starting point was the founding narrative behind urban ecology, according to which the city was absent from ecology until the 1970s. We felt there was value in examining the hypothesis of a regime of imperceptibility of the city in ecology by looking at its spatial and historical contexts.

We found inspiring theoretical resources in agnotology and in the concept of the knowledge blind spot, which we felt offered promising avenues for capturing an absence of the city which did not seem to be the result of a deliberate strategy. The collateral nature of this absence made a head-on approach to the problem impractical. We therefore sought to address the city’s imperceptibility to ecological science by adopting a multi-faceted approach, focusing on global science but also on the more situated production of knowledge through a case study in Switzerland.

We submit that the lack of references to the city in the ecological literature can be considered an indirect effect of dominant epistemic framings, field practices and institutional marginality. Many strands of ecology cannot conceive of the city as a spatio-epistemic category for structural and constitutive reasons. The ways in which spatio-epistemic processes that characterize knowledge production (De Bont and Lachmund, 2017) hindered the constitution of urban sites as meaningful places and objects for ecological science (see also Gieryn, 2006) thus represent a key dimension of this regime of imperceptibility.

By focusing on the Swiss case, we have shown that local research practices do not always reflect international research agendas in terms of priorities and timelines. Ecological research on the city failed to emerge for a number of reasons: because it was conducted on the margins of academe or at the boundaries of established professions, because scientific entrepreneurship and material resources were weak and because it did not fit with dominant research agendas and disciplinary cultures. Our Swiss case study invites us to attend to the contextualized and contingent histories of missed opportunities, marginal initiatives and precarious researchers that characterize ordinary science in the (non)making.

Exploring the regime of imperceptibility of the city in ecology is a fruitful way to nuance and deepen the contours of the history of urban ecology. Drawing continuities and discontinuities with the dominant narratives of urban ecology, our results highlight the existence of ecological research related to the city *before* and *alongside* what goes by that label.

Our in-depth examination of the historical (dis)connections between amateur naturalist practices in the city and the emergence of specific currents of academic and professionalized urban ecology invites us to look beyond the linear genealogies that assume continuity between the two. The decentred perspective adopted here, from within Switzerland but situating it in networks of knowledge circulation, enabled us to bring to light the geography of a research field. It allowed us to reveal how agendas and practices that have been successfully developed elsewhere – in our case, by the Berlin School of Urban Ecology – can materialize in marginal local configurations. We contend that the dominant historiography of urban ecology can be enriched and extended by looking at it from the margins of its European traditions.

Finally, adopting a relational perspective that sees urban ecological research as fundamentally embedded in broader disciplines and adjacent fields, we have highlighted how the growing interest in anthropized landscapes such as cities is closely related to the environmental turn and the emergence of activist pro-conservation scholarship that shaped the ecological sciences beginning in the 1970s. These shifts prompted the development of theoretical and methodological approaches at the crossroads of different disciplines, such as landscape ecology and conservation biology, with which urban ecology research has aligned itself and from which it has benefitted. We have shown that, in some local contexts, the emergence of urban ecology research as part of this movement has taken dissident and alternative forms which, having neither institutional existence nor autonomy, remained invisible to the disciplinary core of ecology.

We hope our contribution can expand the critical dialogue with insiders’ historiography and narratives on urban ecology. The motif of the city’s absence has been strategically employed by scholars aspiring to foster a community and establish a distinct academic field. We have shown that not all research, and not all researchers, were able or motivated to be involved in such a venture, or to embrace the kind of commitment and scientific entrepreneurship it implies. The creation of new scientific fields, or the fragmentation of existing ones, and the demarcation operations in both form and substance that they entail generate imperceptibility. They require forms of research that may appear similar, but that do not correspond exactly to the project being pursued, to be made invisible or their marginality to be exaggerated. Ignorance studies have largely been developed with a view to exposing biased historiographies, or the post-truth politics at work behind health or environmental scandals. Here, we have hypothesized and attempted to illustrate that this analytical framework can be fruitfully used to address the invisibility and marginality of prior and competing research in the formation and consolidation of novel epistemic communities. Mostly used to denounce unequal power relations, it could also be mobilized as a reflexive tool to address the constitution of novel scientific fields, especially in a context of global ecological crisis and scholarly activism where narratives, symbols and performances hold a privileged position in scientists’ claims.

## Supplemental Material

sj-docx-1-epf-10.1177_26349825241241522 – Supplemental material for Ecology’s inattention to the city: Exploring a regime of scientific imperceptibility Supplemental material, sj-docx-1-epf-10.1177_26349825241241522 for Ecology’s inattention to the city: Exploring a regime of scientific imperceptibility by Maud Chalmandrier, Valérie Boisvert, Joelle Salomon Cavin, Silvia Flaminio and Céline Granjou in Environment and Planning F

## Notes

1. ‘When Ecology Goes to Town’, 23–25 January 2019, University of Lausanne.

2. Our research focuses on both ‘city’ and the ‘urban’ in ecology, following how actors use, label and define their research objects across time and space. Yet, we chose ‘the city’ as the umbrella term for the purposes of the general argument of the article, for two reasons. The first is that our historical approach (from the end of the 19th century) also covers a period when the ‘urban’ did not exist as a category, and the process of ‘urbanization’ was not yet conceptualized as such, especially in European cities. The second is due to cultural-linguistic differences: Compared with the prominent use of the ‘urban’ in English, in German, interest in the city in ecological research has primarily been expressed using terms beginning with the stem ‘*Stadt*’ (city): *Stadtflora*, *Stadtökologie.*

3. The 26 cantons are the member states of the Swiss Confederation.

4. https://www.e-periodica.ch/. E-Periodica is an open-access platform hosted by the ETH Zurich library which compiles digitized articles published in Swiss journals from the 18th century to the present day.

5. Information on this example comes from the annual activity reports of the Geobotanical Institute from 1973 to 1998 (*Berichte des Geobotanischen Instituts der Eidg. Techn. Hochschule, Stiftung Rübel*; available at www.e-periodica.com) and from interviews.
