# Supplementary material for: Ecology’s inattention to the city: Exploring a regime of scientific imperceptibility
Source: Environ Plan F Philos Theory Models Method Pract. 2024 Apr 22;4(4):470–91. doi: 10.1177/26349825241241522 (PMC13044429; doi:10.1177/26349825241241522)
Supplement: sj-docx-1-epf-10.1177_26349825241241522 – Supplemental material for Ecology’s inattention to the city: Exploring a regime of scientific imperceptibility [file sj-docx-1-epf-10.1177_26349825241241522.docx]

**Methodological appendix: constituting the corpus of Swiss naturalist societies publications referring to the city**

Source: <https://www.e-periodica.ch/>. Platform hosted by the library of ETH Zurich, which lists digitized articles of Swiss journals covering the period from the 18^th^ century to the present day. The platform was chosen for its rigorous referencing, the quality of the scans, and the availability of advanced searches by keyword in the body of the text.

**Figure 1: Journals of Swiss naturalist societies selected in the corpus via the online open-access platform e-periodica**


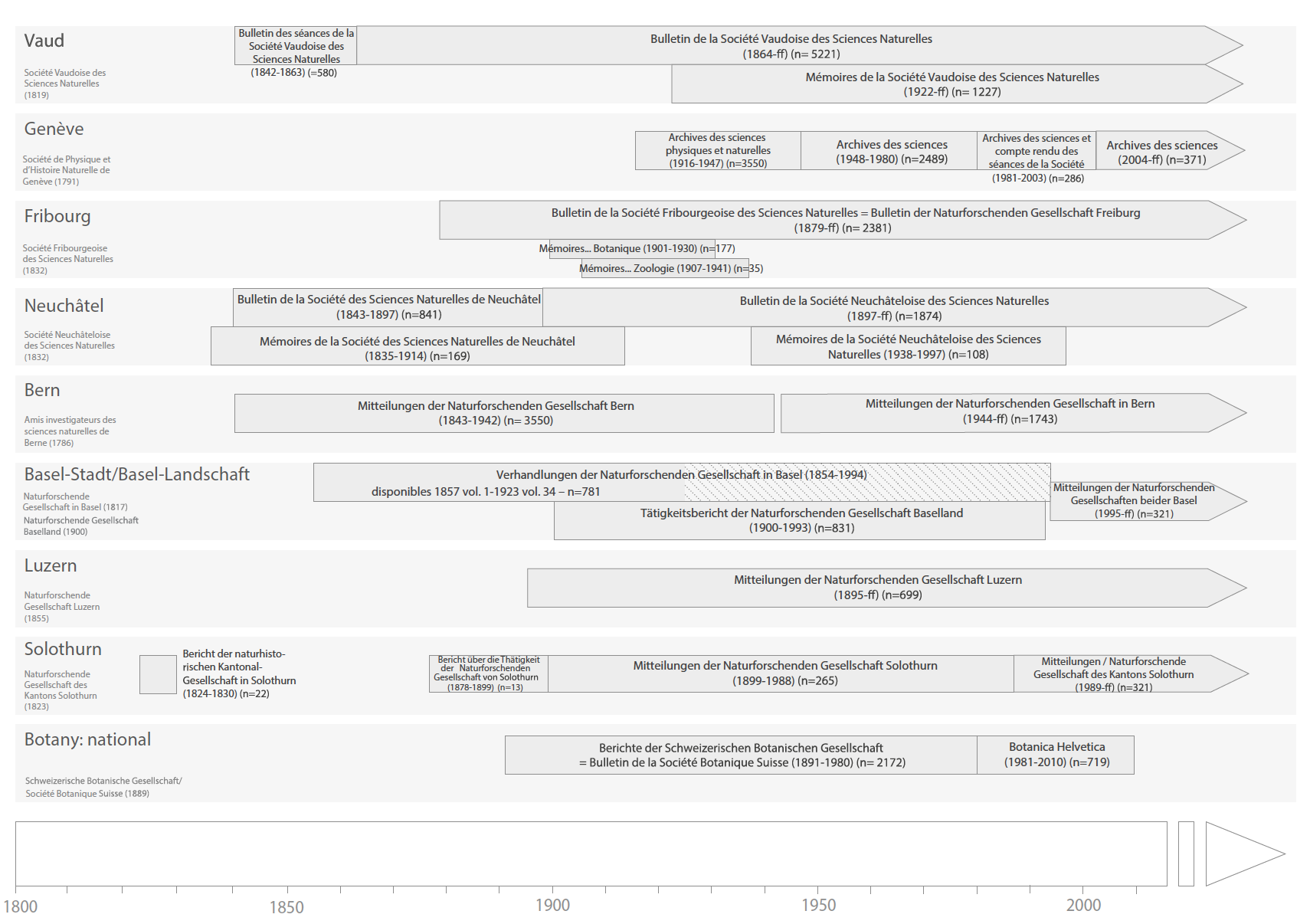


**Figure 2: Publication selection and encoding steps**

| **Steps** | | **Selection criteria** | | | | | **Number after selection** |
| --- | --- | --- | --- | --- | --- | --- | --- |
| **1** | **Trilingual search on the open access platform e-periodica** | In the whole text, for any of these word forms: | stadt*, städt*, ville*, agglo*, urbain*, urban*, city, cities | | | 1599 | |
|  |  | Excluded | Publications in physics, chemistry, geology. | | |  |  |
|  | | | | | | | |
| **2** | **Cumulative selection criteria** | Ecological or naturalistic dimension | Included | Study of non-human living organisms (fauna/flora/mycetes), which corresponds globally to research in botany, zoology, mycology and entomology. | 445 | | |
|  |  |  | Borderline cases, included | Studies on climate, pollution, lake and water management when they integrate a biotic element (indicator species, impact on fauna or flora); archaeozoology; microbial ecology. |  |  |  |
|  |  |  | Borderline cases, excluded | Palaeontology; bacteriology. |  |  |  |
|  |  | Relation with the city | Included | Fieldwork at least partially located in the city or its immediate vicinity (near the city); at least one species survey located in the city; discourse on nonhuman beings in the city, the city, or urbanization without necessarily involving urban field research (or any fieldwork). |  |  |  |
|  |  |  | Borderline cases, included | Lake and water management studies when located in cities or if a link with urbanization is explained. |  |  |  |
|  |  |  | Excluded | City as a biographical element or institutional address; studies on nature reserves located near the city but where the functional link with the city is not made. |  |  |  |
|  | | | | | | | |
| **3** | **Coding** | Object of study | Fauna | Studies on animals, sometimes with specification of taxa ([in]vertebrates, birds, mammals, insects, etc.). | 152 | | |
|  |  |  | Flora | Studies on plants, sometimes with specification of taxa (vascular plants, trees, lichens, etc.). For simplicity, mushrooms, which are in fact fungi, were included in this group. | 237 | | |
|  |  |  | Fauna/flora | Studies on animals and plants. | 35 | | |
|  |  |  | Other | Publications that do not specifically deal with fauna or flora (landscape, conservation, etc.). | 21 | | |
|  |  |  | | | | | |
|  |  | Location of field research | City 1 | Publications whose field research or object of study is at least partly located in the city. | 289 | | |
|  |  |  | City 2 | Publications whose field research or object of study is located in the immediate surroundings of the city, according to its author(s). | 142 | | |
|  |  |  | | | | | |
|  |  | Scale of analysis | Local | Publications that focus on a city, a portion of a city, or a localized case study. | 141 | | |
|  |  |  | Regional | Publications whose study perimeter is the canton or a region defined by its author(s). | 210 | | |
|  |  |  | National | Publications covering Swiss territory as a whole. | 59 | | |
|  |  |  | | | | | |
|  |  | Centrality of the city as object | Object | Publications with fieldwork conducted at least partly in cities or their immediate surroundings, and discourse on the city, nonhuman beings in cities, or urbanization. Publications explicitly on urban ecology are included in this category. | 91 | | |
|  |  |  | Discourse | Publications containing general discourse on the city or urbanization without involving field research or empirical study of the city. | 102 | | |
|  |  |  | Inventory | Publications, mainly inventories, featuring fieldwork or species collected at least partly in the city, without including discourse on the city, nonhuman beings in cities, or urbanization. Inventories including these types of discourse were assigned to the ‘Object’ category, which means that species inventories are divided between the two. | 245 | | |
|  |  |  | Other | Publications that do not fit into any of the above categories. | 6 | | |
|  |  |  | | | | | |
|  |  | Type of publication | Article | Publications of a study, inventory, or an observation note. | 382 | | |
|  |  |  | Book | Books or catalogues published as an entire issue of the journal. One book = one article. | 7 | | |
|  |  |  | Book chapter | Chapter of a book published in a journal as a special issue. One chapter corresponds to one article. | 14 (4 books) | | |
|  |  |  | Activity report | Activity reports of naturalist societies’ specialized committees (e.g. Commission of Nature Protection), associations (Pro Natura), or related societies (local botanical society), annual activity reports of naturalist societies (including information on conferences, excursions, etc.). | 30 | | |
|  |  |  | Bibliography | Book reviews or bibliographic lists (e.g. lists of annual publications in Swiss botany). | 5 | | |
|  |  |  | Necrology | Obituary in homage to a member of the naturalist society. | 3 | | |
